# Tomoelastography based on multifrequency MR elastography predicts liver function reserve in patients with hepatocellular carcinoma: a prospective study

**DOI:** 10.1186/s13244-022-01232-5

**Published:** 2022-06-03

**Authors:** Huimin Lin, Yihuan Wang, Jiahao Zhou, Yuchen Yang, Xinxin Xu, Di Ma, Yongjun Chen, Chunxue Yang, Ingolf Sack, Jing Guo, Ruokun Li, Fuhua Yan

**Affiliations:** 1 Department of Radiology, Ruijin Hospital, Shanghai Jiao Tong University School of Medicine, No. 197 Ruijin Er Road, Shanghai, 200025 China; 2 Department of General Surgery, Ruijin Hospital, Shanghai Jiao Tong University School of Medicine, Shanghai, China; 3 Department of Pathology, Ruijin Hospital, Shanghai Jiao Tong University School of Medicine, Shanghai, China; 4 Department of Radiology, Charité–Universitätsmedizin Berlin, Berlin, Germany

**Keywords:** Tomoelastography, Magnetic resonance elastography, Hepatocellular carcinoma, Liver function reserve, Indocyanine green retention rate

## Abstract

**Background:**

Estimating liver function reserve is essential for preoperative surgical planning and predicting post-hepatectomy complications in patients with hepatocellular carcinoma (HCC). We investigated hepatic viscoelasticity quantified by tomoelastography, a multifrequency magnetic resonance elastography technique, to predict liver function reserve.

**Methods:**

One hundred fifty-six patients with suspected HCC (mean age, 60 ± 1 years; 131 men) underwent preoperative tomoelastography examination between July 2020 and August 2021. Sixty-nine were included in the final analysis, and their 15-min indocyanine green retention rates (ICG-R15s) were obtained to determine liver function reserve. Tomoelastography quantified the shear wave speed (*c*, m/s), which represents stiffness, and loss angle (*φ*, rad), which represents fluidity. Both were correlated with the ICG-R15. A prediction model based on logistic regression for major hepatectomy tolerance (ICG-R15 ≥ 14%) was established.

**Results:**

Patients were assigned to either the ICG-R15 < 14% (*n* = 50) or ICG-R15 ≥ 14% (*n* = 19) group. Liver *c* (*r* = 0.617) and *φ* (*r* = 0.517) were positively correlated with the ICG-R15 (both *p* < 0.001). At fibrosis stages F1–2, *φ* was positively correlated with the ICG-R15 (*r* = 0.528; *p* = 0.017), but *c* was not (*p* = 0.104). At stages F3–4, *c* (*r* = 0.642; *p* < 0.001) and *φ* (*r* = 0.377; *p* = 0.008) were both positively correlated with the ICG-R15. The optimal cutoffs of *c* and *φ* for predicting ICG-R15 ≥ 14% were 2.04 m/s and 0.79 rad, respectively. The area under the receiver operating characteristic curve was higher for *c* (0.892) than for *φ* (0.779; *p* = 0.045).

**Conclusions:**

Liver stiffness and fluidity, quantified by tomoelastography, were correlated with liver function and may be used clinically to noninvasively assess liver function reserve and stratify treatments.

**Supplementary Information:**

The online version contains supplementary material available at 10.1186/s13244-022-01232-5.

## Key points

1. Hepatic biomechanical properties were sensitive to liver function reserve.
2. Liver stiffness and fluidity quantified by tomoelastography could be potential biomarkers for liver function assessments.
3. Liver fluidity showed unique sensitivity to liver function reserve at early fibrosis (F1–2) stage.

## Background

Hepatocellular carcinoma (HCC) is the most common primary malignant liver tumor and ranked as the third leading cause of cancer death worldwide [1]. Hepatectomies are the preferred treatment for patients without cirrhosis, in whom major resection could be performed without life-threatening complications [1, 2]. Cirrhosis results in different degrees of liver injury. For patients with chronic liver diseases or liver cirrhosis, liver function should be assessed to determine the disease severity and make informed decisions for clinical treatment [3]. Preoperative estimation of liver function reserve for surgical planning may help reduce post-hepatectomy morbidity and mortality rates [2].

Child–Pugh classification is the most commonly used method in clinical practice to evaluate liver function and enables partial evaluation of the surgical risk [4]. Some institutions consider Child–Pugh grade A to be a surgical indication for hepatectomy [2, 5]. Indocyanine green (ICG) elimination is an effective dynamic test for describing liver function before liver surgery [6, 7]. The 15-min ICG retention rate (ICG-R15) is the most widely used parameter to estimate hepatocyte function, and its values are approximately < 10% in normal persons [8]. Elevated retention rates may reflect a reduced ability of the liver to regenerate after resection and an increased risk of postoperative hepatic failure [9]. Various confounding factors and the crudeness and subjectivity of the clinical indicators limit the performance of the Child–Pugh classification system [10]. Patients at Child–Pugh grade A can exhibit ICG-R15s ranging from 5.6 to 32.0% [5, 11]. Patients with an ICG-R15 of < 14% generally tolerate major hepatectomies well, whereas as those with an ICG-R15 > 20% should not undergo major liver resection [2]. The ICG retention test is considered the most precise method for assessing liver function reserve [6].

With the development of imaging technology, several recent studies have used ultrasound elastography to measure liver function reserve. Liver stiffness enables quantifying liver fibrosis of mixed etiologies to various degrees [12, 13]. As a common outcome of various hepatic diseases, liver cirrhosis, developed from liver fibrosis, is characterized by the accumulation of extracellular matrix (ECM) proteins and formation of fibrous scarring, leading to replacement and distortion of hepatic parenchymal tissue, thus resulting in loss of liver function [14]. Studies have found that liver stiffness measured by transient elastography (TE) [15], point shear wave elastography (pSWE) [16], and acoustic radiation force impulse (ARFI) elastography [17, 18] are well correlated with liver function test results, such as Child–Pugh grades or ICG-R15, but with varying correlation coefficients ranging from 0.342 to 0.862. Wei et al. [18] reported that the area under the curve (AUC) of the ARFI for diagnosing patients who were at least Child–Pugh class B was 0.841 (95% confidence interval [CI] 0.756–0.905). In a recent study, liver stiffness quantified by 2D time-harmonic elastography was also found to be correlated with liver function as measured by the 13C-methacetin Liver MAximum capacity test during fibrogenesis [19]. Research in this area suggested that ultrasound-based liver elasticity may be a supplementary indicator for assessing liver functional reserves and could provide valuable prognostic information for patients undergoing resection.

Magnetic resonance elastography (MRE) is a noninvasive imaging technique for quantifying the biomechanical properties of tissue [20, 21]. MRE has been shown to be valuable in detecting and staging liver fibrosis [22–25], predicting portal hypertension [26–28], and diagnosing and characterizing tumor invasiveness [29–32]. Similar to studies using ultrasound [16, 17], a previous two-dimensional (2D)-MRE study of 32 patients with HCC first reported that liver stiffness of the non-tumor parenchyma was significantly and positively correlated with the ICG-R15, and MRE parameters may potentially effectively assess the liver function reserves of these patients [33]. Compared with ultrasound, MRE has a wider measurement range using 2D or 3D liver tissue maps, which could help overcome the sampling error that occurs with ultrasound [21]. MRE is also suitable for patients who are obese and/or have ascites and has higher repeatability in clinical applications. However, research on the efficiency of MRE to evaluate liver function remains scarce. To our knowledge, there was one MRE study on liver function reserve [33]. This study was limited in sample size, and only liver stiffness was investigated.

Tomoelastography, an advanced multifrequency MRE technique, is an emerging noninvasive imaging modality used to characterize biomechanical properties of the tissue. With multifrequency data acquisition and wavenumber-based inversion method, the parameter maps provided by tomoelastography can reveal rich anatomical details [34]. Tomoelastography provides two viscoelastic parameters for biomechanical characterization of soft tissues [35]: shear wave speed (*c*, m/s) and loss angle of the complex shear modulus (*φ*, rad), which are surrogate indicators of stiffness and viscosity (or fluidity), respectively, through postprocessing. Tomoelastography has been applied for the biomechanical characterization of a variety of diseases in vivo, including pancreatic diseases [30, 36], neuro-tumors [37], prostate diseases [35, 38], rectal carcinoma [32], inflammatory bowel disease[39], liver tumors [29, 40], and chronic liver disease [41, 42]. Because microstructural properties in the ECM and cell conditions alter liver fibrosis progression, we hypothesized that stiffness and fluidity, mostly associated with the ECM constituents, could indicate hepatic pathological processes and may reflect liver function [43].

Thus, in this study, we aimed to (1) investigate correlations between the liver mechanical properties derived from tomoelastography and liver function reserve evaluated by the ICG test and (2) develop a tomoelastography prediction model of liver function reserve for patients with a low tolerance for major liver resection.

## Material and methods

### Study sample

**Fig. 1:**
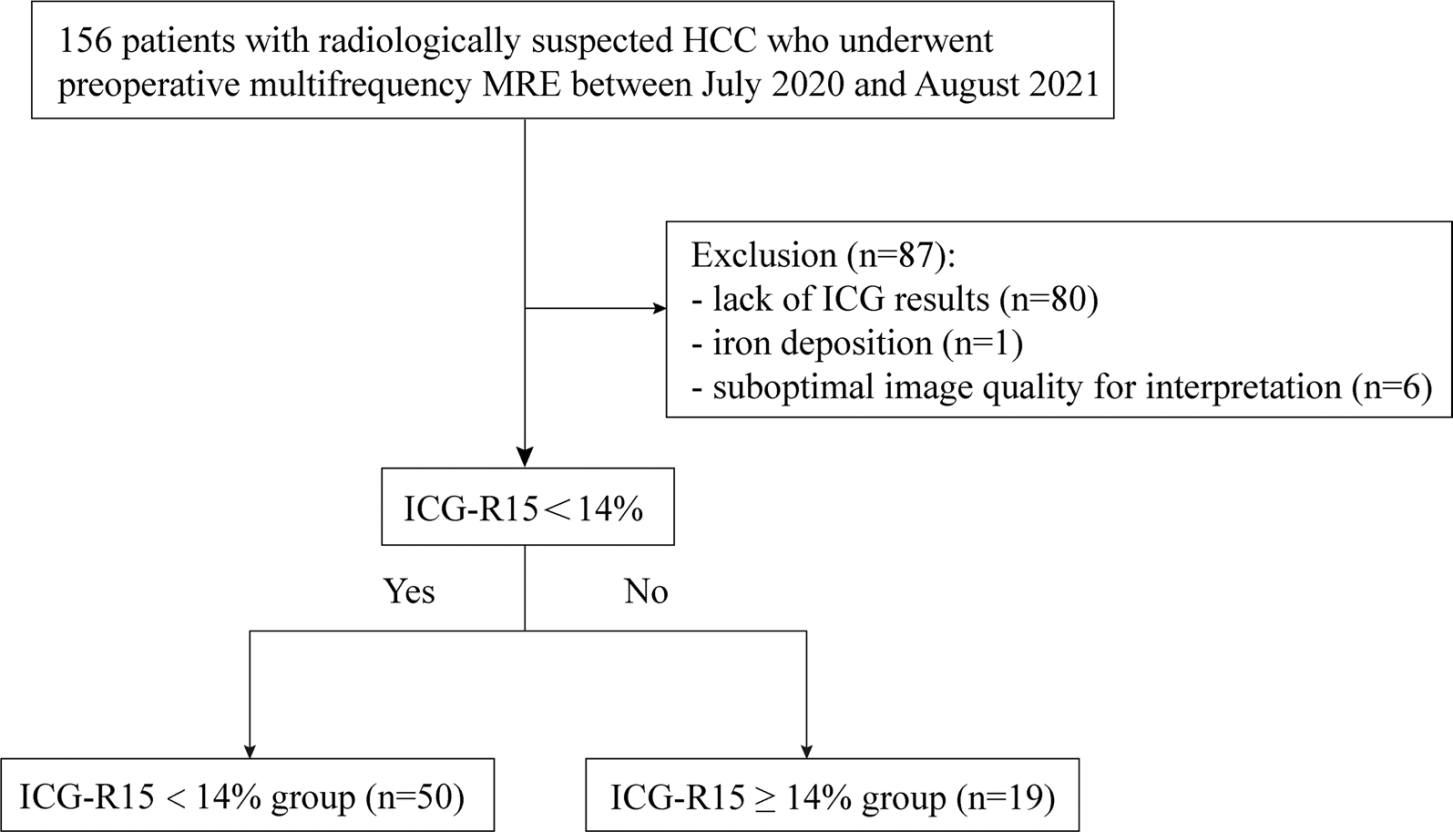
Flow diagram of the study participants. HCC: hepatocellular carcinoma, ICG: indocyanine green, ICG-R15: 15-min ICG retention rate

The Ruijin Hospital institutional review board approved this prospective study (No. RJ2018-209), and all participants provided written informed consent. From July 2020 to August 2021, 156 patients with radiologically suspected HCC, who underwent preoperative tomoelastography, were enrolled in this prospective study. The exclusion criteria were (a) lack of ICG results, (b) iron deposition, and (c) suboptimal image quality on tomoelastography. Eighty-seven patients were excluded, and 69 were included in the final analysis (mean age, 58 ± 10 years; 56 men, 13 women). Based on their ICG-R15 values, the final 69 patients were categorized into either the ICG-R15 < 14% (*n* = 50; mean age, 58 ± 10 years; 45 men, 5 women) or ICG-R15 ≥ 14% (*n* = 19; mean age, 59 ± 9 years; 11 men, 8 women) group (Fig. 1).

### Tomoelastography

Tomoelastography examinations were performed with a 1.5T scanner (MAGNETOM Aera, Siemens, Erlangen, Germany). The setup was similar to that described in Shahryari et al. [29]. Briefly, mechanical vibrations of 30, 40, 50, and 60 Hz were generated and transferred sequentially to the liver, using four pressure pads driven by compressed air. Two anterior and two posterior pads, operating at 0.4 and 0.6 bar, respectively, were placed near the liver region. The 3D wave field was acquired using a single-shot, spin-echo echo-planar imaging sequence with flow-compensated motion encoding gradients (MEG). Fifteen consecutive transverse slices with a field of view (FoV) of 384 × 312 mm^2^ (matrix size 128 × 104) and 3 × 3 × 5 mm^3^ resolution were acquired during free breathing. Additional imaging parameters included echo time (ET) = 59 ms; repetition time (TR) = 2050 ms; parallel imaging with GRAPPA factor 2; MEG frequencies of 43.48 Hz for the 30, 40- and 50 Hz vibration frequencies and 44.88 Hz for the 60 Hz vibration frequency; and a MEG amplitude of 30 mT/m. The total acquisition time was approximately 3.5 min.

Multifrequency wave field data were processed using the processing pipeline available at https://bioqic-apps.com. Full FoV high spatial resolution maps of shear wave speed (*c*) and phase angle (*φ*) were generated. Because *c* is proportional to the square root of the storage modulus and *φ* continuously changes from 0 (solid properties) to π/2 (viscous properties), they are considered surrogates for stiffness and fluidity, respectively. Throughout the text, we use *c* and *φ* to provide quantitative information and “stiffness” and “viscosity” (or “fluidity”) to discuss qualitative changes.

Regions of interest (ROIs) were manually drawn using tomoelastography magnitude images to encompass as much of the background liver as possible on three consecutive sections with the largest liver cross-sectional coverage on the central *c*- and *φ*-map slices. The measurements were averaged and used as the representative parameters. Two radiologists (rater #1 with 8 years of experience and rater #2 with 1 year of experience) analyzed the tomoelastography data independently for all patients to test interobserver variability.

### Clinical liver function test

The ICG clearance test was performed before surgery and within 1 week of tomoelastography scanning. After fasting for 6 h, ICG was injected through a peripheral venous access at 0.5 mg/kg body weight, and the injection was completed within 10 s. ICG-R15s were registered via a digital pulse densitometer, which was connected to a DDG-3300 K device (Pulsion Medical Systems, Nihon Kohden, TYO, JP) through a near-infrared finger piece sensor. ICG-R15 values < 14% were considered safe for major resection [2], and we used this value to stratify patients. Information on hepatic encephalopathy and ascites as well as total bilirubin (TB), albumin (ALB), prothrombin time (PT), increased international normalized ratio (INR), prealbumin (PAB), platelet (PLT), aspartate aminotransferase (AST), and alanine aminotransferase (ALT) values was collected before surgery. Child–Pugh grades were assessed using the Child–Pugh scoring system [4], and patients were classified as class A: 5–6; class B: 7–9; or class C: 10–15.

### Histopathological analysis

Liver tissue samples were obtained via surgical resection. Liver fibrosis grades were analyzed by a pathologist with 10 years of experience in hepatic pathology, who was blinded to all radiological and clinical results.

### Statistical analysis

The chi-square test was used to compare qualitative parameters between two groups. Student’s t test or the Mann–Whitney *U* test were used for quantitative data. Interobserver agreement was analyzed for biomechanical parameters by using intraclass correlation coefficients (ICCs). Pearson rank correlation was performed to analyze the relationships between normally distributed variables; Spearman correlation was used for non-normally distributed variables. Receiver operating characteristic (ROC) curves were used to analyze the diagnostic efficacy of the parameters, and area under the ROC curves (AUROCs) was calculated with 95% confidence intervals (CIs). All statistical analyses were performed with SPSS software (version 26, SPSS for Windows, IBM, Armonk, NY, USA), GraphPad Prism software (version 8.0, GraphPad Prism for Windows, La Jolla, CA, USA), and MedCalc software (MedCalc Software, Ltd., Solvusoft, LV, NV, USA). Two-tailed *p* values < 0.05 were considered statistically significant.

## Results

### Demographics of the study population

We included 69 patients with HCC in our study. Table 1 summarizes their demographic characteristics. Compared with the ICG-R15 < 14% group, the ICG-R15 ≥ 14% group had a higher proportion of women (*p* = 0.007), lower PAB (*p* < 0.001) and PLT (*p* = 0.002) levels, and increased PT (*p* < 0.001) and INR (*p* < 0.001) levels. Liver *c* (1.81 ± 0.29 vs. 2.39 ± 0.45, respectively, *p* < 0.001) and *φ* (0.75 ± 0.11 vs. 0.86 ± 0.11, respectively, *p* = 0.001) values differed significantly between the ICG-R15 < 14% and ICG-R15 ≥ 14% groups. No other demographic or laboratory results differed significantly between the groups.

**Table 1:** Demographic and clinical characteristics of the ICG-R15 < 14% and ICG-R15 ≥ 14% groups

Characteristic	ICG-R15 < 14% group (n = 50)	ICG-R15 ≥ 14% group (n = 19)	*p* value
Patients			
Age, years (range)	58 ± 10 (38–81)	59 ± 9 (38–74)	0.746
Sex (male/female)	45:5	11:8	*0.007
BMI (kg/m^2^)	23.5 ± 2.9	24.2 ± 3.2	0.378
Etiology (%)			0.3923
Hepatitis B virus	38 (76.0)	17 (89.4)	
Hepatitis C virus	3 (6.0)	1 (5.3)	
Other	9 (18.0)	1 (5.3)	
Laboratory results			
ALB (g/L)	40.04 ± 4.18	35.89 ± 9.49	0.080
PAB (mg/L)	205.88 ± 51.68	117.63 ± 45.19	* < 0.001
TB (μmol/L)	19.67 ± 12.56	22.26 ± 9.75	0.420
AST (U/L)	28 (23, 48.5)	38 (28, 61)	0.090
ALT (U/L)	28 (20, 49.25)	26 (19, 36)	0.282
PLT (× 10^9^/L)	144.68 ± 55.43	97.00 ± 49.91	*0.002
PT (s)	12.24 ± 0.92	13.98 ± 1.37	* < 0.001
INR	1.04 ± 0.08	1.19 ± 0.12	* < 0.001
MRE parameters			
Liver* c* (m/s)	1.81 ± 0.29	2.39 ± 0.45	* < 0.001
Liver *φ* (rad)	0.75 ± 0.11	0.86 ± 0.11	*0.001

ICG-R15: 15-min indocyanine green retention rate, BMI: body mass index, ALB: albumin, PAB: prealbumin, TB: total bilirubin, AST: aspartate aminotransferase, ALT: alanine aminotransferase, PLT: platelet, PT: prothrombin time, INR: international normalized ratio

*p < 0.05

Sixty-eight patients were categorized as Child–Pugh class A, of whom 47 had a score of 5 and 21 had a score of 6. The remaining patient, who had a score of 8, was categorized as class B owing to their biochemical data and clinical symptoms. In the Child–Pugh class A patients, the ICG-R15s ranged from 1.3 to 48%; the patient in class B had an ICG-R15 of 20.7% (Table 2).

**Table 2:** ICG-R15 ranges by Child–Pugh grade

Child–Pugh Grade	Score	*n*		ICG-R15 (%) (range)
A	5	47	68	1.3–48
	6	21		
B	8	1	1	20.7

ICG-R15: 15-min indocyanine green retention rate

ICG-R15, liver *c* and *φ* were significantly higher in participants with higher fibrosis grades of F3–4 than in those with liver fibrosis grades of F1–2 (Table 3). Additionally, the F3–4 group had lower PAB (*p* = 0.021) and PLT (*p* < 0.001) values and increased PT (*p* = 0.042) and INR (*p* = 0.039) values.

**Table 3:** ICG-R15 and tomoelastography indexes between patients with different liver fibrosis grades

Liver fibrosis frade	F1–2 (*n* = 21)	F3–4 (*n* = 48)	*p* value
ICG-R15, % (range)	4.9 (1.8–14.0)	9.2 (1.3–48.0)	*0.005
Liver			
* c* (m/s)	1.64 ± 0.14	2.13 ± 0.42	* < 0.001
* φ* (rad)	0.70 ± 0.07	0.82 ± 0.12	* < 0.001
Age, years (range)	58 ± 12 (38–81)	58 ± 9 (38–74)	0.937
Sex (male/female)	18:2	31:10	0.325
BMI (kg/m^2^)	24.1 ± 3.3	23.5 ± 2.9	0.501
Laboratory results			
ALB (g/L)	39.29 ± 4.57	38.73 ± 6.98	0.739
PAB (mg/L)	208.05 ± 60.8	170.00 ± 61.85	*0.021
TB (μmol/L)	16.39 ± 4.50	22.13 ± 13.56	0.066
AST (U/L)	26.0 (23.5, 42.5)	35.5 (24.5, 56.8)	0.196
ALT (U/L)	30.0 (19.5, 60.0)	25.5 (20.0, 41.3)	0.393
PLT (×10^9^/L)	172.25 ± 53.32	111.39 ± 49.03	* < 0.001
PT (s)	12.24 ± 1.09	12.94 ± 1.36	*0.042
INR	1.04 ± 0.09	1.10 ± 0.12	*0.039

ICG-R15: 15-min indocyanine green retention rate, BMI: body mass index, ALB: albumin, PAB: prealbumin, TB: total bilirubin, AST: aspartate aminotransferase, ALT: alanine aminotransferase, PLT: platelet, PT: prothrombin time, INR: international normalized ratio

*p < 0.05

### ICCs

**Fig. 2:**
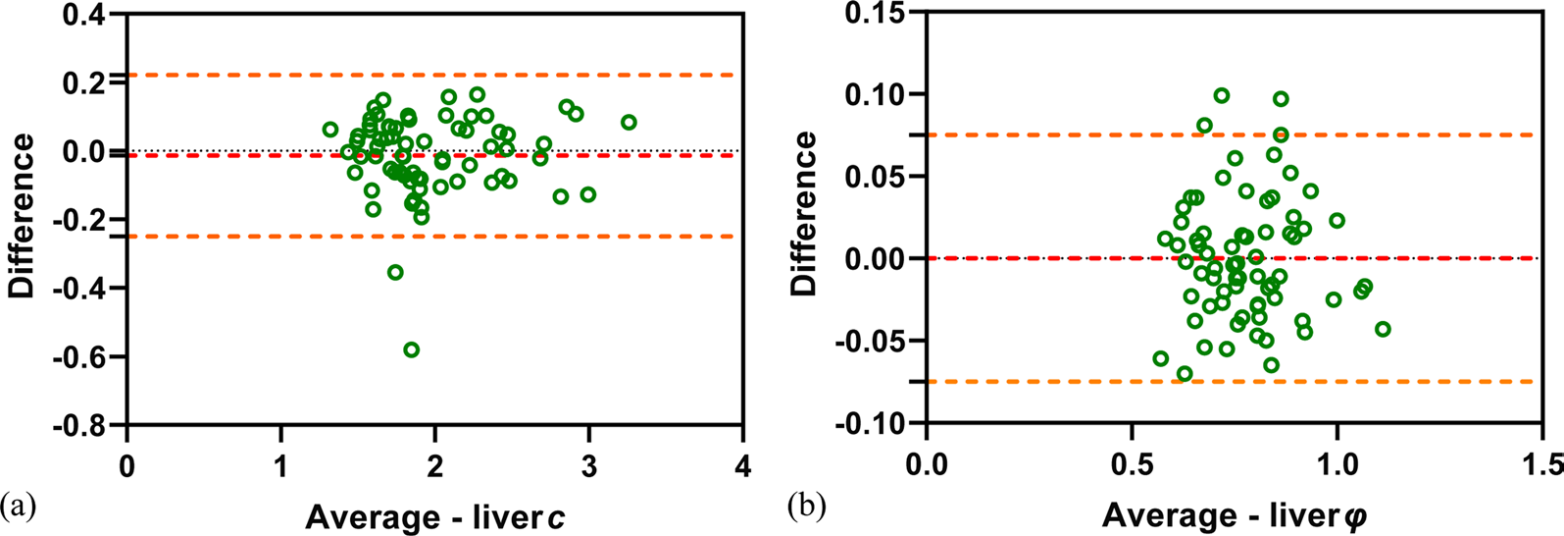
Bland–Altman plots showing agreement between liver *c* and *φ* values evaluated by two independent raters

The ICCs, representing interobserver reliability of the mechanical properties for all patients evaluated by two raters, were 0.960 (95% CI 0.936–0.975) for *c* and 0.949 (95% CI 0.920–0.968) for *φ*, suggesting good concordance and data consistency (Fig. 2).

### Correlation analysis of biomechanical parameters and clinical tests

**Fig. 3:**
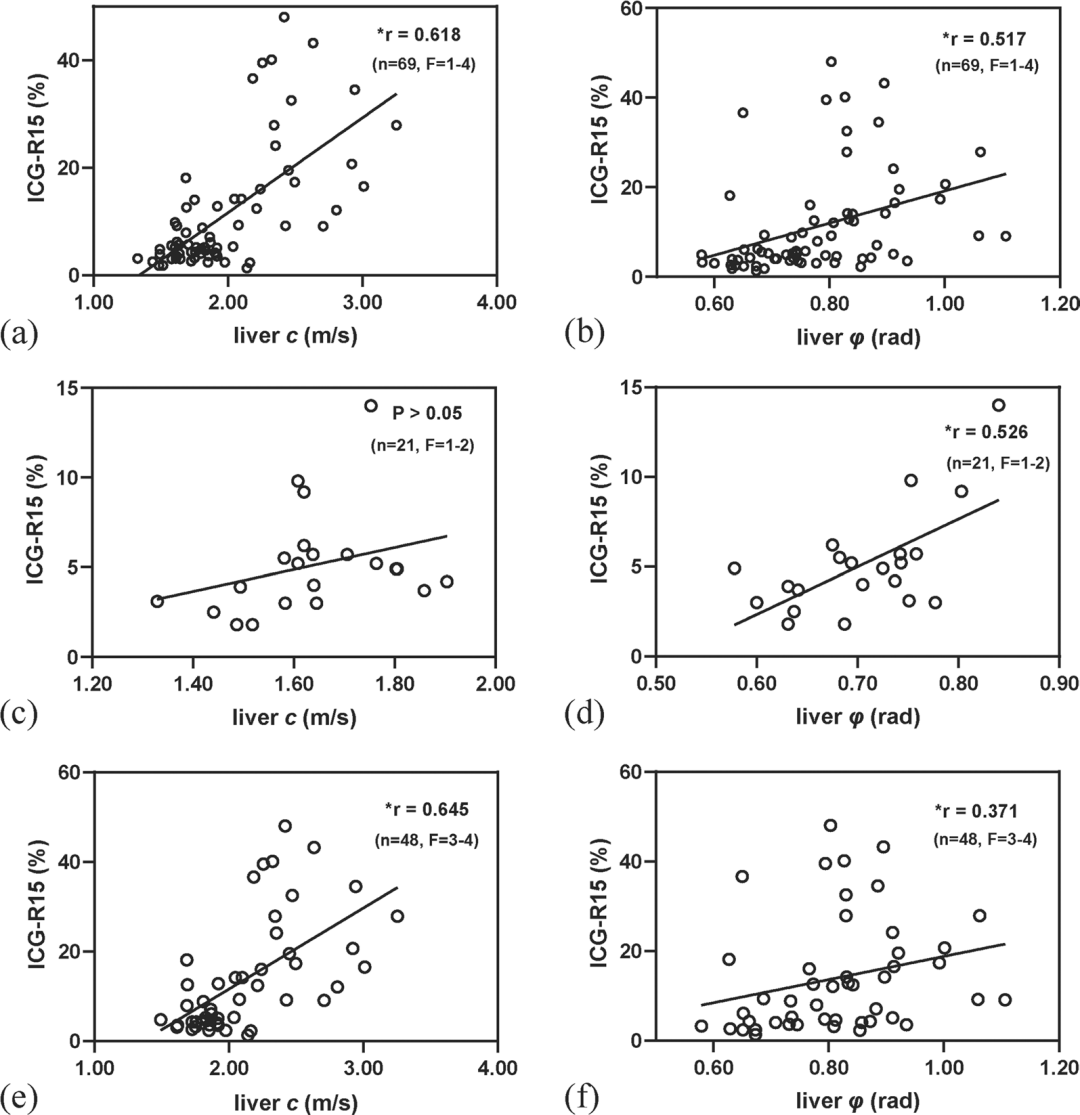
Scatterplots showing that liver *c* (*r* = 0.618; *p* < 0.001) (**a**) and liver *φ* (*r* = 0.517; *p* < 0.001) (**b**) were positively correlated with the ICG-R15. In liver fibrosis stages F1–2, liver *φ* (*r* = 0.526; *p* = 0.014) was positively correlated with ICG-R15 (**d**), but liver *c* (*p* = 0.109) was not (**c**). In liver fibrosis stages F3–4, liver *c* (*r* = 0.645; *p* < 0.001) (**e**) and liver *φ* (*r* = 0.371; *p* = 0.009) (**f**) were positively correlated with the ICG-R15. *p < 0.05. ICG-R15: 15-min indocyanine green retention rate

**Fig. 4:**
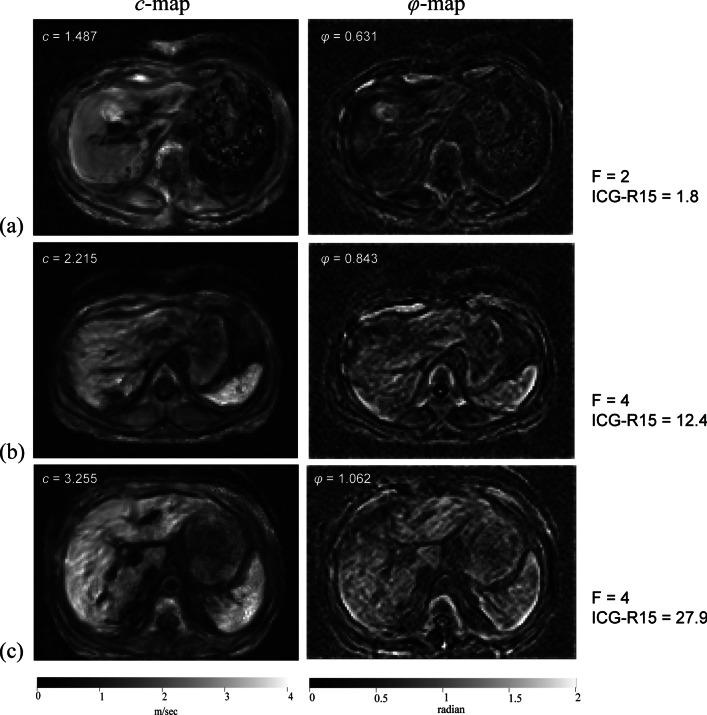
Axial tomoelastography *c* and *φ* maps of the ICG-R15 data and fibrosis stages for three patients. **a** *F* = 2; *c*, 1.487 m/s; *φ*, 0.631 rad; ICG-R15, 1.8; **b** *F* = 4; *c*, 2.215 m/s; *φ*, 0.843 rad; ICG-R15, 12.4; **c** *F* = 4; *c*, 3.255 m/s; *φ*, 1.062 rad; ICG-R15, 27.9. ICG-R15: 15-min indocyanine green retention rate

Blood samples were taken from all patients. TB, AST, PT, and INR were positively correlated, and PLT, ALB, and PAB were negatively correlated with both liver *c* and *φ* (Table 4). Liver *c* (*r* = 0.617) and *φ* (*r* = 0.517) were positively correlated with the ICG-R15 (both *p* < 0.001; Fig. 3). In liver fibrosis stages F1–2, liver *φ* (*r* = 0.528; *p* = 0.017) was positively correlated with the ICG-R15, but liver *c* (*p* = 0.104) was not. In liver fibrosis stages F3–4, liver *c* (*r* = 0.642; *p* < 0.001) and liver *φ* (*r* = 0.377; *p* = 0.008) were positively correlated with the ICG-R15. Figure 4 shows the axial tomoelastography *c* and *φ* maps for three patients with their corresponding ICG-R15 data and fibrosis stages.

**Table 4:** Pearson or Spearman correlation between blood index and tomoelastography parameters

			PLT		TB	ALB	PAB	AST	ALT	PT	INR
Liver *c* (m/s)		*r*		− 0.388	0.397	− 0.302	− 0.528	0.366	0.036	0.491	0.493
		*p*		*0.001	*0.001	*0.012	* < 0.001	*0.002	0.770	* < 0.001	* < 0.001
Liver *φ* (rad)		*r*		− 0.344	0.183	− 0.306	− 0.259	0.367	0.208	0.317	0.316
		*p*		*0.004	*0.132	*0.011	*0.032	*0.002	0.086	*0.008	*0.008

PLT: platelet, TB: total bilirubin, ALB: albumin, PAB: prealbumin, AST: aspartate aminotransferase, ALT: alanine aminotransferase, PT: prothrombin time, INR: international normalized ratio

*p < 0.05

### Predictive performance of tomoelastography in predicting ICG-R15 ≥ 14%

**Fig. 5:**
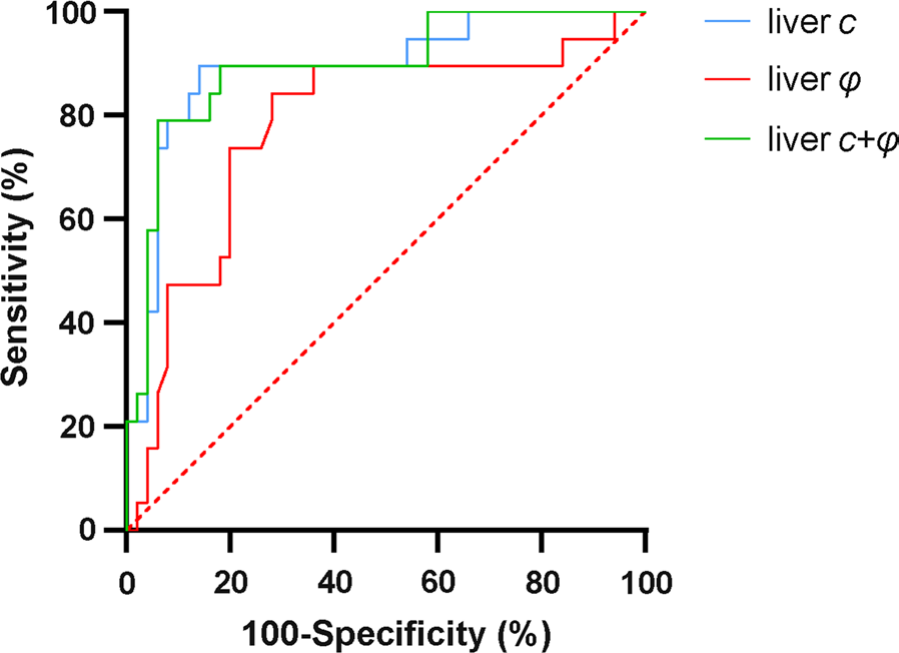
Liver *c*, liver *φ* and their combination for detecting an ICG-R15 > 14%. ICG-R15: 15-min indocyanine green retention rate

The optimal liver *c* and *φ* cutoff values for predicting the ICG-R15 ≥ 14% group were 2.04 m/s and 0.79 rad, respectively. The AUROC was higher for *c* than for *φ* (0.892, 95% CI 0.793–0.954 vs. 0.779, 95% CI 0.663–0.870; *p* = 0.045). However, combining *c* and *φ* did not significantly increase the AUROC compared with that of *c* alone (Table 5, Fig. 5).

**Table 5:** Performances of the models in predicting ICG-R15 > 14%

Models	Cutoff value	AUROC	*p* value	Sensitivity (%)	Specificity (%)
*c* (m/s)	2.04	0.892 [0.793–0.954]	…	89.5 (17/19) [66.9–98.7]	86.0 (43/50) [73.3–94.2]
*φ* (rad)	0.79	0.779 [0.663–0.870]	*0.045	84.2 (16/19) [60.4–96.6]	72.0 (336/50) [57.5.9–83.8]
*c* + *φ*	…	0.895 [0.797–0.956]	0.702	79.0 (15/19) [54.4–93.9]	94.0 (47/50) [83.5–98.7]

Data in parentheses are numerators/denominators; data in brackets are the 95% confidence intervals. AUC of the combined *c* and *φ* was obtained by using probabilities estimated from logistic regression. AUC values were compared using the Delong test with respect to an AUC value of *c*

ICG-R15: 15-min indocyanine green retention rate, AUROC: area under receiver operating curve

*p < 0.05

## Discussion

Preoperatively assessing liver function reserve is critical for surgical planning and predicting prognoses. Therefore, a noninvasive and quantitative biomarker is needed to accurately determine liver function. In the current study, we investigated the mechanical manifestation of liver function reserve using in vivo tomoelastography in a group of patients with HCC. We demonstrated the sensitivity of two biomechanical parameters corresponding to tissue stiffness and fluidity quantified by tomoelastography for predicting insufficient liver function reserve. In our patient cohort, diminished liver function was correlated with increased hepatic stiffness and fluidity. Moderate correlations between tomoelastography parameters and serum markers revealed that biomechanical parameters were indicative of liver function. Associations between hepatic stiffness and liver function reserve have been reported previously [15–18, 33, 44] (Additional file 1: Table S1) and were largely explained by excessive deposition of ECM elements during fibrogenesis. This caused liver lobule structural changes, hepatocellular injuries, and altered hepatic vascular structure and resistance, thus disrupting and compromising liver function.

Fluidity, another biomechanical parameter recovered from tomoelastography, was introduced for the first time in this study to assess liver function reserve. Similar to liver stiffness, liver fluidity was positively correlated with the ICG-R15, demonstrating that livers with low function reserves behaved more fluidlike. Reiter et al. [45] reported increased liver fluidity with advanced fibrotic stages, which was attributed to elevated mechanical friction resulting from dissociations of ECM proteins, possible tissue compression, and development of regenerative nodules. Interestingly, we found that fluidity was uniquely sensitive to liver function reserves in the early fibrosis (F1–2) group, whereas stiffness was not. As collagen deposition alone is likely insufficient to cause vascular structure alterations during early fibrosis [46], liver stiffness relating to collagen content might be nonresponsive to ICG-R15. However, the progression of inflammation was more prominent than collagen accumulation in the F1–2 group. Inflammation-associated increases in vascular permeability and vascular leakage could result in excessive fluid permeating the vessel wall and a consequent increase in internal tissue friction [47, 48], which explains the elevation in ICG retention and liver fluidity as well as the positive correlation between the two.

Our study had limitations. First, this was a single-center study. A multicenter study is warranted with hospitals where tomoelastography is availability. Second, our sample size was relatively small, especially in the F1–2 group. A large cohort study is planned to further validate our preliminary findings. Third, the HCC samples in our study were from fibrotic/cirrhotic livers of different etiologies. Although most of our patients had liver cirrhosis associated with chronic hepatitis, a more defined liver background with similar pathogeneses would allow more accurately identifying liver function predictors. Finally, the scope of our study did not cover the postsurgical outcome assessment which is of high interest and relevance. This aspect will be incorporated in our future studies to further validate the performance of tomoelastography in assessing liver function.

## Conclusions

In conclusion, in vivo tomoelastography enabled quantitatively measuring hepatic biomechanical properties that were sensitive to liver function reserves. Liver fluidity showed unique sensitivity to liver function reserve at early fibrosis (F1–2) stage which might be associated with inflammation. Liver stiffness and fluidity may be potential biomarkers for noninvasively assessing liver function to allow making informed treatment decisions. Future studies in large patient cohort with postoperative follow-ups are warranted.

## Supplementary Information

**Additional file 1. Table S1.** Characteristics of the related works. AUROC: area under receiver operating curve, TE: transient elastography, HCC: hepatocellular carcinoma, LS: liver stiffness, ICG-R15: 15-min indocyanine green retention rate, ARFI: acoustic radiation force impulse, MRE: magnetic resonance elastography, HBV: hepatitis B virus.

## Data Availability

The datasets used and/or analyzed during the current study are available from the corresponding author on reasonable request.

## Abbreviations

ALB: Albumin
ALT: Alanine aminotransferase
ARFI: Acoustic radiation force impulse
AST: Aspartate aminotransferase
AUROC: Area under ROC
BMI: Body mass index
CI: Confidence interval
ECM: Extracellular matrix
ET: Echo time
FoV: Field of view
HCC: Hepatocellular carcinoma
ICC: Intraclass correlation coefficients
ICG-R15: Indocyanine green retention rate at 15 min
INR: International normalized ratio
MEG: Motion encoding gradients
MRE: Magnetic resonance elastography
PAB: Prealbumin
PLT: Platelet
pSWE: Point shear wave elastography
PT: Prothrombin time
ROC: Receiver operating characteristic
ROIs: Regions of interest
TB: Total bilirubin
TE: Transient elastography
TR: Repetition time
