# Supplementary material for: Tomoelastography based on multifrequency MR elastography predicts liver function reserve in patients with hepatocellular carcinoma: a prospective study
Source: Insights Imaging. 2022 Jun 3;13:95. doi: 10.1186/s13244-022-01232-5 (PMC9166923; doi:10.1186/s13244-022-01232-5)
Supplement: Supplementary file 1 — Additional file 1. Table S1. Characteristics of the related works. AUROC: area under receiver operating curve, TE: transient elastography, HCC: hepatocellular carcinoma, LS: liver stiffness, ICG-R15: 15-min indocyanine green retention rate, ARFI: acoustic radiation force impulse, MRE: magnetic resonance elastography, HBV: hepatitis B virus. [file 13244_2022_1232_MOESM1_ESM.docx]

**ELECTRONIC SUPPLEMENTARY MATERIAL**

**Tomoelastography Based on Multifrequency MR Elastography Predicts Liver Function Reserve in Patients with Hepatocellular Carcinoma: A Prospective Study**

**Supplemental Table 1.** Characteristics of the related works

| Author, year | Technique | Sample size | Etiology | Correlation between | Correlation coefficient | AUROC in predicting | AUROC |
| --- | --- | --- | --- | --- | --- | --- | --- |
| Fung J, 2013 [15] | TE | 44 | HCC | LS and ICG-R15 | 0.342 | / | / |
| Sun XL, 2015 [17] | ARFI | 76 | Liver tumors | LS and ICG-R15 | 0.862 | / | / |
|  |  |  |  | LS and Child-Pugh grade | 0.772 | / | / |
| Li B, 2015 [33] | MRE | 32 | HCC | LS and ICG-R15 | 0.746 | / | / |
| Wei L, 2020 [18] | ARFI | 104 | HBV-related cirrhosis | LS and Child-Pugh grade | 0.457 | decompensated cirrhosis | 0.841 |

AUROC: area under receiver-operating curve, TE: transient elastography, HCC: hepatocellular carcinoma, LS: liver stiffness, ICG-R15: 15-min indocyanine green retention rate, ARFI: acoustic radiation force impulse, MRE: magnetic resonance elastography, HBV: hepatitis B virus
